# Factors associated with institutional delivery practice among women in pastoral community of Dubti district, Afar region, Northeast Ethiopia: a community-based cross-sectional study

**DOI:** 10.1186/s12978-019-0782-x

**Published:** 2019-08-13

**Authors:** Wassie Sadik, Alemayehu Bayray, Ayal Debie, Tsegaye Gebremedhin

**Affiliations:** 1 Family Health Coordinator, Afar Regional Health Bureau, Afar, Ethiopia; 2 Department of Epidemiology and Biostatistics, Mekelle University, Mekelle, Ethiopia; 3 Department of Health Systems and Policy, Institute of Public Health, College of Medicine and Health Sciences, University of Gondar, Po. Box: 196, Gondar, Ethiopia

**Keywords:** Institutional delivery, Practice, Pastoral community, Dubti, Ethiopia

## Abstract

**Background:**

Eighty-five percent of the global burden of maternal mortality was covered by Sub-Saharan Africa. Ethiopia is a major contributor to the death of mothers with a maternal mortality ratio of 676 per 100,000 live births. Only 10% of deliveries in Ethiopia were at health facility with the least (6.4%) in the Afar region. However, there is limited evidence about factors of institutional delivery in the study area. Thus, this study aimed to assess the magnitude and associated factors of institutional delivery practice among women in the pastoral community of Dubti district, Northeast Ethiopia.

**Methods:**

A community based cross-sectional study was conducted from April to May 2016, in the pastoral community of Dubti district. A total of 381 women were selected using systematic sampling technique and interviewed using a standardized structured questionnaire. Binary logistic regression analysis was computed. In the final multivariable logistic regression analysis adjusted odds ratio (AOR) with 95% confidence interval (CI) was used to declare the factors associated with institutional delivery.

**Results:**

This study revealed that 35.2% (95% CI: 30.5–40.1) of women were delivered at the health facility. Women who had travelled less than an hour to reach the nearest health facilities (AOR: 4.90, 95% CI: 2.62–9.18), attending antenatal care (AOR: 2.50, 95% CI:1.48–4.23), previous history of stillbirth (AOR: 4.34, 95% CI: 1.78–10.58), good knowledge (AOR: 2.09, 95% CI:1.23–3.56), and husband involved in decision making on delivery place (AOR: 4.42, 95% CI: 1.98–9.90) were the factors associated with institutional delivery practice.

**Conclusions:**

The overall institutional delivery practices in the study area was low as compared to the national level. This low practice of institutional delivery was contributed by residing far from the facility, does not received antenatal care, and having low awareness about ANC follow up and institutional delivery services. Therefore, strengthening the accessibility of health facility to nearby mothers resided, antenatal care services, and awareness creation provision at the community level for pregnant women in the pastoral community can improve institutional delivery practice.

## Plain English summary

Institutional delivery is the corner stone to reduce maternal death and improve neonatal health. The practice of institutional delivery services utilization is very low in the pastoral community of Dubti district. One of the major contributors to high maternal deaths in Ethiopia was low institutional delivery. Accordingly, low practice of institutional delivery in the study area could also affect the health status and wellbeing of the newborn. Moreover, there are number of factors that hinder institutional delivery service utilization in the community. Those factors might be linked with women’s cultural, social, and personal factors.

In the study area, nothing is known about the factors that affect institutional delivery service practices. Therefore, this study aimed to identify the factors that hinder institutional delivery service practices among women who gave birth in the last 12 months. Structured interviewer-administered questionnaire was used for interviewing the women. Accordingly, women who had resided away from the health facility might not receiving follow up and did not face any complication during the recent pregnancy. Additionally, they did not know the benefit of institutional delivery service and had no any involvement of their husband on decision of delivery place were factors associated with institutional delivery utilization of the women.

## Background

Childbirth is an essential affair in the lives of families and the building of communities. Almost all fatal outcomes and disabling result of women during childbirth can be prevented and much of the suffering can be alleviated with skilled and responsive care at delivery and during the postnatal period [1]. An estimated 287,000 maternal deaths occurred worldwide in 2011. From this, Sub-Saharan Africa accounts for 56% and Southern Asia 29% which is constituted for 85% of the global burden of maternal death [2]. Globally, 80% of all maternal deaths occurred due to direct causes which are hemorrhage, hypertensive disorders, infections, unsafe abortion, sepsis, and obstructed labor [3].

Complications during pregnancy and childbirth are the leading causes of death among adolescent girls aged 15–19 years in low and middle-income countries resulting in thousands of deaths each year [4, 5]. Maternal death is high particularly in settings with high home delivery, and areas with poor civil registration systems to identify the correct causes of maternal death [6]. Ethiopia was one of the leading contributors to maternal deaths in Sub-Saharan African countries. With maternal mortality ratio (MMR) of 676 per 100,000 live births in 2011 [7]. The Ethiopian Mini Demographic and Health Survey (EMDHS), 2014 report indicated that institutional delivery in Afar region was 6.4% which was the lowest among the nine regions in Ethiopia [8]. Furthermore, 19% of women had four or more antenatal care (ANC) visits during their recent pregnancy and only 10% of pregnant women delivered at the health facility [8] which indicates low utilization of modern maternal health care.

Every pregnancy faces a risk which may not always be detected through the risk assessment approach during ANC follow up. However, the majority of deaths due to obstetric complications are preventable and the best way to assure a safe and successful delivery outcome was through attending institutional delivery with a skilled birth attendant. But it was not well known why the majority of pastoral pregnant women did not deliver at the health institution. Therefore, this study was designed to assess the magnitude and associated factors of institutional delivery practice among women who gave birth in the last 12 months in the pastoral community of Dubti district, Afar region, Northeast Ethiopia.

## Methods

### Study design and setting

A community based cross-sectional study design was conducted at Dubti district in Afar Regional State from April to May, 2016. The district has 14 (2 urban and 12 rural) kebeles (the smallest administrative unit). It has also 1 referral hospital, 3 health centers, and 10 Health posts. The source population were all reproductive age group women who gave birth within the last 12 months in Dubti district, whereas the study population was all reproductive age group women who gave birth within the last 12 months in the selected kebeles of Dubti district.

### Sample size and sampling techniques

The sample size was calculated by using both single and double population proportion formula. The sample size for the first objective was calculated by using single population proportion formula with an assumption of proportion (P) =16.7% [9], 4% margin of error (d), 95% confidence level, and 10% non-response rate. *N* = (Z_α/2_) [2]*P (1-P)/(d) [2] = (1.96)^2^(0.167) (0.833)/(0.04) [2] = 334. The final sample size for the first objective was 368.

Additionally, the sample size for the second objective was calculated by using double population proportion formula considering 80% power, 95% confidence level, 10% non-response rate. The proportion of institutional delivery among ANC attended women (30.7%) [10] and among urban residents (22.4%) [11] among women who gave birth at health facility had taken for sample size calculation. Then the calculated sample size by using were 376 and 383, respectively. The calculated sample size for the second factor in the double population proportion formula was higher than others. Therefore, for maintaining sample size adequacy we used 383 which was the largest. Then, one urban and three rural kebeles among 14 kebeles of the district were selected using simple random lottery method. The sample was proportionally allocated to each selected kebeles based on their total estimated birth rate. Proportionate stratified systematic sampling technique was used to select the participants. The first woman was selected through simple random lottery method. Then, every other household woman starting from the first selected household were used to select the study participant. Accordingly, when two or more women who had lived together within a household had got, one of them was selected through simple random lottery method.

### Measurement and variables

A woman who gave birth for their last child at health institution for the last 1 year were considered as institutional delivery. Knowledge of women was measured by using 8 item questions each containing (0 = No and 1 = yes) alternatives and those women who scored 60% and above the total knowledge measuring score were considered as having good knowledge. Accordingly, the attitude of women towards institutional delivery was measured by using 6 item questions each containing (1 = strongly disagree, 2 = disagree, 3 = neutral, 4 = agree, and 5 = strongly agree) alternatives and those women who scored 60% and above the total attitude measuring score were considered as having a favorable attitude.

### Data collection tools and procedures

An interviewer-administered structured questionnaire was adapted from different studies [11–14] and used to collect data among women aged 15–49 years who gave birth in the last 12 months. The questionnaire was initially developed in English and translated into local Afaraff language and finally back to English in order to ensure its consistency. Four diploma clinical nurses for data collectors and two bachelor degree nurses for supervisors were recruited. The training was given for 1 day on the basic techniques of data collection and pre-testing was conducted on 19 women at Logia district (neighboring district which has similar population characteristics and infrastructure to Dubti district) before the actual data collection. Finally, all findings from the pre-test were incorporated into the final questionnaire. During data collection, supervisors have checked the data for accuracy, consistency, and completeness on a daily basis.

### Data management and analysis

Data were cleaned, coded and entered to EPI info version 3.5.1 and exported to STATA version 13 software for analysis. Frequencies and percentage of variables were presented using tables and graphs. Bivariable and multivariable logistic regression analysis were conducted and those predictor variables with *P*-value less than 0.2 in the bivariable logistic regression analysis were entered to multivariable logistic regression analysis. Adjusted Odds Ratio (AOR) with 95% CI was used to identify the strength and associated factors with the outcome variable. Those independent variables with a *P*-value of less than 0.05 during multivariable logistic regression analysis were considered as significantly associated with the outcome variable.

## Results

### Socio-demographic and economic characteristics

A total of 381 reproductive age group women who gave birth in the last 12 months had participated in the study. The mean age (± SD) of the study participants was 25.7 (± 5.3) years. More than three-fourth (86.3%) of the participants were Muslim followers. Out of all participants, 91.6% were married. Furthermore, 62.9% of the participants’ occupation was staying at home (housewives) and 47.8% of their husband’s occupation was pastoralist. Accordingly, 68.2% of participants were Afar in their ethnicity and 64.3% were not educated. About 23.9% of women had less than 500 Ethiopian birr ($17.8) monthly household income (Table 1).

**Table 1:** Socio-demographic and economic characteristics of participants in Dubti district, Afar region, Northeast Ethiopia, 2016 (*n* = 381)

Variables	Category	Frequency (n)	Percent (%)
Age in years	15–19	45	11.81
	20–24	133	34.91
	25–29	111	29.13
	30–34	65	17.06
	> 35	27	7.09
Marital status	Married	349	91.60
	Divorced	19	4.99
	Single	13	3.41
Residence	Urban	96	25.20
	Rural	285	74.80
Occupation of women	Pastoral	70	18.37
	Merchant	37	9.71
	Government employee	34	8.92
	Stay at home	240	62.99
Religion	Christian	52	13.65
	Muslim	329	86.35
Ethnicity	Afar	260	68.24
	Oromo	13	3.41
	Amhara	94	24.67
	Tigray	14	3.68
Monthly income	< 500 ETB	91	23.88
	500–1000 ETB	160	41.99
	> 1000 ETB	130	34.12
Women literacy status	Not educated	245	64.30
	Able to read and write	48	12.60
	Primary education	52	13.65
	Secondary education	13	3.41
	Higher education	23	6.04
Husband literacy status	Not educated	150	42.98
	Able to read and write	104	29.80
	Primary education	29	8.31
	Secondary education	37	10.60
	Higher education	29	8.31
Occupation of husband	Farmer	58	16.62
	Pastoralist	167	47.85
	Merchant	79	22.64
	Government employee	45	12.89

*ETB* Ethiopian Birr

### Obstetric history of the study participants

In this study, 42.7% of women had attended at least one antenatal visit during their recent pregnancy. Of those, only 23.3% of them had four or more antenatal visits and more than half (55.2%) had been started their first antenatal visit during their third trimester. More than 20 % of women had five or more history of pregnancy and delivery. Similarly, nearly 20 and 10% of women had previous history of abortion and stillbirth, respectively. Furthermore, 41.7 and 46.2% of women had good knowledge and favorable attitude towards institutional delivery, correspondingly (Table 2).

**Table 2:** Knowledge of women on institutional delivery in Dubti district, Afar region, Northeast, Ethiopia, 2016 (*n* = 381)

Variables	Category	Frequency (n)	Percent (%)
Attend ANC	No	218	57.22
	Yes	163	42.78
Number of ANC visit (*n* = 163)	One	71	43.56
	Two-three	54	33.13
	>Four	38	23.31
Gestational age during 1st visit (*n* = 163)	1st trimester	33	20.25
	2nd trimester	40	24.54
	3rd trimester	90	55.21
Gravidity	One	60	15.75
	Two to four	299	60.10
	> Five	92	24.15
Parity	One	92	24.15
	Two – four	205	53.81
	> five	84	22.05
Number of under five children	One	93	24.41
	>Two	288	75.59
Total family size in a household	<Three	98	25.72
	Three to four	82	21.52
	> five	201	52.76
History of abortion	No	307	80.58
	Yes	74	19.42
Frequency of abortion (*n* = 74)	One	68	91.89
	>Two	6	8.11
History of still birth	No	344	90.29
	Yes	37	9.71
Frequency of still birth (*n* = 37)	One	32	86.49
	>Two	5	13.51
Knowledge of women	Good	159	41.73
	Poor	222	58.27
Attitude of women	Favorable	176	46.19
	Unfavorable	205	53.81
History of difficult labor	No	264	69.29
	Yes	117	30.71
Travel time to reach the HF	≤1 h	205	53.81
	> 1 h	176	46.19
Source of information (*n* = 362)	Health workers	285	78.73
	Mass media	77	21.27

*HF* Health Facility

### Institutional delivery practice of participants

Overall 35.2% (95% CI: 30.5–40.1%) of women had gave birth at the health facility. From women who gave birth at the health facility, for 45.5% women who had gave birth at health facility had been attended by midwiferies followed by nurses 25.4%. Forty six percent of the husband of women were involved in the decision of delivery place. On top of this, 20.6% of women reported that they gave birth at home since the health facilities were too far from their home. About one-fourth (24.3%) of women who had delivered at home were attended by their mother in law. Twenty percent of women who had delivered at health facility reported that institutional delivery was one of the major factors that contribute for saving maternal life. About 32 % of women had faced complication during their recent birth. Nearly two-thirds (65.3%) of women had excessive vaginal bleeding among those who had faced complications (Table 3).

**Table 3:** Recent institutional delivery practice of mothers in Dubti district, Afar region, Northeast Ethiopia, 2016 (*n* = 381)

Variables	Category	Frequency (n)	Percent (%)
Institutional delivery	No	247	64.8
	Yes	134	35.2
Who assisted the delivery at HF? (*n* = 134)	Nurse	34	25.37
	Health officer	12	8.96
	Midwifery	61	45.52
	Doctor	27	20.15
Who was decision-maker for the place of delivery?	Herself	66	17.32
	Husband	175	45.93
	Other relatives	140	36.75
Who assisted home delivery? (*n* = 247)	Her mother	46	18.62
	Mother in law	60	24.29
	TTBA	51	20.65
	Neighbor	54	21.87
	Health Extension Workers	36	14.57
Reasons for HF delivery (*n* = 134)	Received health education	38	28.36
	Good service	69	51.49
	Save mothers life	27	20.15
Have you faced any complication during your recent labor and delivery (381)	Yes	121	31.8
	No	260	68.2
Type of complication that faced during labor and delivery? (*n* = 121)	Excessive Vx bleeding	79	65.29
	Retained placenta	8	6.61
	Prolonged labor	22	18.18
	Mal presentation	12	9.92
Reasons for home delivery (*n* = 247)	Fast labor	26	10.53
	Transport problem	45	18.22
	Health facility far	51	20.65
	Husband refuse	40	16.19
	Fear of user fee	21	8.50
	Poor service provision	14	5.67
	Feel shame	21	8.50
	Others	29	11.74

### Factors associated with institutional delivery

In the multivariable logistic regression analysis, women traveling within an hour or less far from the nearby health facilities were 4.90 times more likely to deliver at health institution than women traveling more than an hour (AOR: 4.90, 95% CI: 2.62–9.18). Women who attend ANC during their recent pregnancy were 2.50 times more likely to attend institutional delivery as compared to their counterparts (AOR: 2.50, 95% CI: 1.48–4.23). Those mothers having a history of stillbirths were 4.34 times more likely attending intuitional delivery compared to those mothers who had no history of stillbirth (AOR: 4.34, 95% CI: 1.78–10.58). Women having good knowledge about delivery and pregnancy-related complications and services were 2.09 times more likely to deliver at a health facility than women who had poor knowledge (AOR: 2.09, 95% CI: 1.23–3.56). Moreover, women’s whose husband involved in decision making on the place of delivery were 4.42 times more likely attending institutional delivery compared to women who decide their place of delivery by themselves (AOR: 4.42, 95% CI: 1.98–9.90) (Table 4).

**Table 4:** Logistic regression analysis on factors associated with institutional delivery practice in Dubti district Afar Region, Northeast, Ethiopia, 2016 (*n* = 381)

Variables	Category	Institutional Delivery		COR (95% CI)	AOR (95% CI)
		Yes	No		
Age in years	15–19	11	34	1	1
	20–24	42	91	1.43 (0.66–3.09)	1.73 (0.67–4.46)
	25–29	47	64	2.27 (1.04–4.94)	2.32 (0.80–6.72)
	30–34	26	39	2.04 (0.89–4.78)	1.74 (0.50–6.04)
	> 35	8	19	1.30 (0.45–3.79)	1.20 (0.24–6.14)
Residence	Urban	49	47	2.45 (1.53–3.94)	1.16 (0.60–2.23)
	Rural	85	200	1	1
Women literacy status	Not educated	80	165	1	1
	Able to read & write	18	30	1.24 (0.65–2.35)	1.45 (0.67–3.12)
	Primary	20	32	1.29 (0.69–2.39)	1.34 (0.62–2.88)
	Secondary	5	8	1.28 (0.41–4.07)	2.62 (0.55–12.41)
	Higher	11	12	1.89 (0.80–4.47)	4.33 (0.83–22.61)
Travel time to reach the HF	<1 h	103	102	4.72 (2.94–7.59)	4.90 (2.62–9.18) *
	> 1 hs	31	145	1	1
ANC visit at least once for their recent pregnancy	No	56	162	1	1
	Yes	78	85	2.65 (1.72–4.09)	2.50 (1.48–4.23) *
Gravidity	One	15	45	1	1
	Two to Four	82	147	1.67 (0.88–3.19)	2.18 (0.67–7.07)
	> Five	37	55	2.02 (0.98–4.14)	6.56 (0.76–56.87)
History of abortion	No	107	200	1	1
	Yes	27	47	1.07 (0.63–1.82)	1.14 (0.47–1.89)
History of stillbirth	No	112	232	1	1
	Yes	22	15	3.04 (1.52–6.08)	4.34 (1.78–10.58) *
Knowledge of women	Good	70	89	1.94 (1.27–2.98)	2.09 (1.23–3.56) *
	Poor	64	158	1	1
Attitude of women	Favorable	69	107	1.39 (0.91–2.12)	1.22 (0.73–2.05)
	Unfavorable	65	140	1	1
Decision on delivery place	Herself	12	54	1	1
	Husband	80	95	3.79 (1.90–7.57)	4.42 (1.98–9.90) *
	Other relatives	42	98	1.93 (0.94–3.97)	2.00 (0.87–4.60)

*statistically significant at *p*-value <0.05

## Discussion

Having safe and skilled delivery is one of the cornerstones able to reduce maternal death and improve child health. This study was designed to assess magnitude and determinants of institutional delivery service utilization among women who gave birth in the last 12 months in the pastoral community of Dubti district Northeast, Ethiopia. About 35.2% (95% CI: 30.4–40) of women delivered at the health facility. This result was in line with the studies conducted in Dembecha district, Northwest Ethiopia shows that 34% of mothers delivered at the health facility and in Ghana shows that 37.5% of women delivered at health facility [15, 16]. However, this study finding was higher than a study conducted in Wukro and Butajira districts 25% [10], Sekela 12.1% [14], Munisa 12.3% [11], Dodota 18.2% [17], mini EDHS 2014 report in Afar region 6.4% and nationally 16% [18], EDHS 2016 report of Somali region 18% and Afar region 15%, and nationally in Ethiopia 26% [19], nomads of Sudan 20% [20]. But, this result was lower than a study conducted in Woldia, Ethiopia 48.3% [21], Bahir Dar, Ethiopia 78.8% [13], Tigray Region 59%, Dire Dawa 59%, and Addis Ababa 79% [7], and Tanzania 74.5% [12]. The possible explanation for this variation was due to the difference in the study area, design and period, and also the economical variation of the study participants.

Institutional delivery service utilization was 2.5 times higher among women attended ANC during their recent pregnancy as compared to women who did not attend ANC. This finding is consistent with studies conducted in Sekela and Goba district [14, 22]. This might be due to the information on the importance of institutional delivery they got during their follow up could influence their decision to deliver at health facilities.

Mothers who were traveling an hour or less to reach the nearest health facility were 4.9 times more likely to attend institutional delivery as compared to mothers who were traveling more than an hour to reach the nearest health facility. This finding was consistent with a study conducted in western Ethiopia and Dembecha district [16, 23].

This might be due to mothers who were resided nearby health institutions might have different access to maternal health services such as maternal service-related health education. In addition, mothers had no shortage of transportation access to attend their deliveries at health facilities at any time whether their labor starts. Furthermore, in the pastoral community most of their husbands and adult male family members were seasonally moving from place to place to fed and to get water for their cattle’s and those mothers who were in labor during this time and resided away from health facility had no support to take them to health facilities.

Those mothers who had the previous history of stillbirth were 4.34 times more likely to attend institutional delivery as compared to mothers who had no history of still birth. This finding is in contrast with finding in Dembecha district which mother who had a history of abortion in their life time were less likely to received institutional delivery [16]. The possible explanation for our finding might be due to mothers who had the previous history of stillbirth might increase their concern for the newborn healthy outcome and other complications for the next delivery. This could enhance their probability of attending continuum of maternal health care services; attending ANC and attending ANC services would also improve their utilization of institutional delivery services.

Women who had good knowledge about pregnancy and delivery related services and complications were 2.09 times more likely to attend institutional delivery as compared to women who had poor knowledge. This finding was in line with the study conducted at Sekela district and western Ethiopia [14, 23]. This might be due to knowledgeable women had high probability of capturing the benefits of attending institutional delivery during their ANC visits counseling and other maternal health-related education. The other possible explanation might be mothers having good knowledge relatively may not be affected by traditional malpractices. For instance; in the pastoral community those mothers who delivered at the home considered as the hero of that community, so mothers had good knowledge about the benefits of institutional delivery does not accept the traditional malpractice. In addition, knowledgeable mothers had also a great influence on their husbands and/or other relatives to take them to a health facility as soon as labor was started.

Women whose husband involved during the decision of delivery place was 4.42 times more likely to deliver at a health facility than women’s husband who were not involved. This study was consistent with the study conducted at Dodota district [17]. This might be due to the dominancy of a husband during decision made in the pastoral community which might increase institutional delivery services.

This study might give an insight for policy makers regarding the factors affecting institutional delivery service utilization in pastoral community. However, this study was not supported by qualitative methods. The findings of this study might be subjected to social desirability bias because the respondents were interviewed by health professionals. Furthermore, women might experience recall bias, particularly on the services they had got during their previous obstetrics such as during ANC visit.

## Conclusions

The study showed that institutional delivery service in the study area was low. Mothers resided 1 h or less far from health facilities, attending ANC, previous history of still birth, having good knowledge on pregnancy and child birth, and husband involved during decision making on institutional delivery were factors associated with institutional delivery service utilization. Therefore, strengthen ANC services, awareness creation provision at the community level, and constructing health facilities to the nearby the residents of the community could improve institutional delivery service utilization.

## Data Availability

Data will be available upon reasonable request from the corresponding author.

## Abbreviations

ANC: Antenatal Care
EDHS: Ethiopia Demographic and Health Survey
EMDHS: Ethiopia Mini Demographic and Health Survey
HIV: Human Immune Virus
MMR: Maternal Mortality Ratio
TTBAs: Trained Traditional Birth Attendants
WHO: World Health Organization
